# Developing Multisensory Approach to the Optical Spectral Analysis

**DOI:** 10.3390/s21103541

**Published:** 2021-05-19

**Authors:** Andrey Bogomolov

**Affiliations:** Laboratory of Multivariate Analysis and Global Modeling, Samara State Technical University, 244 Molodogvardeyskaya Str., 443100 Samara, Russia; a.bogomolov@mail.ru

**Keywords:** optical multisensor system, optical spectroscopy, design of experiment, process analytical technology, milk quality, pharmaceutical production, fermentation process monitoring, tumor diagnostics, environmental monitoring

## Abstract

This article presents an overview of research aimed at developing a scientific approach to creating multisensor optical systems for chemical analysis. The review is mainly based on the author’s works accomplished over the recent 10 years at Samara State Technical University with broad international cooperation. It consists of an introduction and five sections that describe state of the art in the field of optical sensing, suggested development methodology of optical multisensor systems, related aspects of experimental design and process analytical technology followed by a collection of practical examples in different application fields: food and pharmaceutical production, medical diagnostics, and ecological monitoring. The conclusion summarizes trends and prospects of the multisensory approach to optical spectral analysis.

## 1. Introduction

The importance of chemical analysis in the modern world is constantly growing. High demands on production efficiency, product quality, and environmental security stimulate the development of instrumental analytical methods. Scientific and technical advances open more and more opportunities for the introduction of analytical control in various areas of human activity: industry, science, medicine, ecology, and even in the personal environment in everyday life.

The analysis of complex multicomponent mixtures is no longer the prerogative of specialized laboratories. To solve modern analytical tasks, new devices are needed that can rapidly monitor the state of the analyzed object on-site, in the production line, or in the field, without permanent sampling. The analysis result should be output without any delay, typically in real time. If necessary, the analyzer must perform tens and hundreds of measurements per second.

The optical methods represent one of the leading directions in the development of a new instrumental base for modern chemical analysis. Currently, this area is experiencing rapid growth, which is largely associated with the technical advances of photonics, namely, with the improvements of existing systems and the emergence of new detection systems, light sources, optics, and light-guiding materials. Optical analysis has a number of practical advantages that include high information content, non-destructiveness, and adaptivity to various objects and media. An important prerequisite for the growing popularity of optical (primarily, spectroscopic) methods is the perfection of information technologies: computers, embedded electronics, and modern methods of multivariate data analysis, also known as chemometrics.

Historically, traditional optical spectroscopy has evolved into a universal analytical solution focused on laboratory measurements of preselected samples of various composition. For this reason, it is badly suited for solving modern analytical problems, such as process monitoring, express analysis, and field research. The low throughput of laboratory spectroscopy does not meet the growing number of analyzed samples and objects to be controlled. The technological complexity of all-purpose spectrometers determines their stationary use and expensiveness, thereby preventing the dissemination of optical spectroscopic methods beyond research institutions, large enterprises, and analytical centers [1].

This situation is starting to change due to the growing development of miniature spectrometers [2,3,4] based on modern sensing and micromechanics technologies, typically having much lower prices compared to the respective lab solutions. The emergence of mini-spectrometers is an important trend in the modern instrumental analytics today that will have an even higher impact in the future. However, in many cases such devices are “semi-finished” products that have signal stability, reproducibility, standardization, and other issues. In practice, mini-spectrometers and similar devices require further development, e.g., adding light sources and measurement interfaces, low-level signal processing, etc., which should be done with a particular application in mind.

Some tasks of operative analytical control of various samples and matrices, especially in the industrial sphere, are successfully solved using simple single-channel sensors, including optical ones. A single-channel optical (photometric) sensor determines the concentration of a component through its correlation with some measurable property of the sample, for example, with the optical density at a selected wavelength. Single-channel sensors are well suited for solving specialized tasks, such as determining the concentration of a suspended component from the medium turbidity, or analyzing samples in which only the determined component gives an optical response. Therefore, the applicability of photometry is limited by the requirement that there are no side factors affecting the measurement. In the quantitative analysis of mixtures, where the analytical signals of various components overlap significantly (which includes the overwhelming majority of real samples), the information content of a single-channel sensor often becomes insufficient and cannot provide the required accuracy of analysis.

In recent years, optical spectroscopy has been developed towards specialized analytical devices intended for specific practical applications. These devices, called *optical multisensor systems (OMS)*, occupy an intermediate position between single-channel photometric sensors and universal laboratory spectrometers. At the same time, they have several striking characteristic features that allow them to be distinguished into a new separate class of optical analyzers. OMS operate in a wide spectral region and can be designed to solve various analytical problems associated with the determination of both individual substances and generalized indicators of chemical composition. Besides, they use a small number of sensory channels, for example, recording the total absorbance at certain wavelength intervals; the lack of selectivity is compensated for by using the mathematical modeling both at the channel optimization stage and in the analysis of measured data. Scientific research in this area and existing implementation examples show the huge potential of OMS. Their further development is expected to bridge the gap between today’s growing demand for analytical control and the limited capabilities of traditional methods. Moreover, this development will help in the formation of new application areas for chemical analysis. Further systematic development and wide dissemination of optical multisensor systems require solving a number of scientific, technical, and methodological problems, which explains the relevance of this work.

The work presented in this paper was generally aimed at developing the scientific foundations of the multisensor approach in optical spectral analysis and the creation of optical multisensor systems for a wide range of analytical applications. To achieve this goal, a number of tasks have been solved. First of all, it was necessary to formulate a general approach to the creation of optical multisensor systems for specific practical applications and to develop new and improved OMS optimization methods and algorithms. Since the optimization is based on the data of a predesigned experiment, it was necessary to systematize the requirements for training and tests samples used in a multicomponent calibration experiment, and to propose an efficient experimental design for building sufficiently accurate application-driven prediction models on a minimum sample set. It was also necessary to develop a methodology for using the multisensory approach in process analytical technology (PAT) to create OMS capable of real-time monitoring of the process state and product quality during production. For the currently working OMS, it was necessary to develop new and improve the existing analytical methods in order to expand the scope and improve the analytical characteristics of such systems. The paper presents selected practical applications of optical multisensor systems in various practical areas including food analysis, pharmaceutical production, biotechnology, medicine, and environmental monitoring. This research was presented on the basis of the author’s habilitation work. The dissertation [5,6] was defended in 2020 at Lomonosov Moscow State University.

## 2. Optical Multisensor Systems in Analytical Spectroscopy: State of the Art

### 2.1. Modern Trends in Optical Spectroscopic Analysis

Optical spectroscopy is one of the most widespread methods of chemical analysis that has a 150-year history [7]. It has traditionally been developed as a universal method for qualitative and quantitative analysis of a wide range of objects. Hence, the prevailing trend is the growing technical complexity of devices to expand their spectral and dynamic ranges, resolution, precision, and reproducibility of analysis. As a result of this development, the modern spectrometers are firmly associated with massive and highly expensive devices used to analyze preliminary acquired samples in centralized laboratories under the supervision of qualified personnel.

An alternative to the traditional spectral analysis began to develop in the last two to three decades. The emerging trends are associated with the fact that the currently dominant laboratory approach does not meet the growing needs of society in chemical analysis. This is primarily due to the constant increase in the number of samples and controlled parameters in existing fields of activity, such as manufacture quality control, environmental monitoring, and medicine. This growth has been accompanied by the emergence of new challenges, areas, and forms of analysis, such as field analysis (also without sampling), in-line analysis in real time, remote analysis (e.g., space measurements), ultra-fast analysis (as in sorting cereals), and distributed sensor networks.

Industrial needs largely dictate the observed conceptual shift for increased product quality requirements, primarily in food and pharmaceutical production. These changes have led to the emergence of a new applied scientific discipline called process analytical technology (PAT) [8,9,10]. The main goal of PAT is to implement the concept of built-in quality, i.e., transition from the output product control to monitoring the process itself [11,12]. Successful introduction of PAT implies, among other requirements, new approaches to data acquisition and analysis, first of all, significantly reducing the time from sample measurements to analysis results. Therefore, the process monitoring should be performed in real time or with an insufficient delay. Depending on the delay, the methods of analysis are usually classified as at-, on-, and in-line ones [12]. Fast and non-destructive optical techniques, including ultraviolet and visible (UV/Vis), near infrared (NIR) [13], IR [14], Raman [15], and fluorescence spectroscopy are considered to be flagship analytical tools of PAT [10].

A separate important trend in the development of analytical spectroscopy is its decentralization and personalization. The emergence of personalized chemical analysis is associated with the expanding range of analyzed objects and its further “democratization” due to the availability, miniaturization, and autonomy of analytical instruments, primarily optical ones. The significant increase in the availability of optical analyzers is going to create new applications and consumer markets. Low-budget analysis can potentially cover the small- and medium-level businesses, become a consumer’s individual tool for product quality control or environmental monitoring, penetrate school education, and fill many other niches. Extrapolating this trend into the future, it is easy to envision a new category of domestic and personal spectral analyzers. The personalization of analysis is greatly facilitated by modern computer development and the wide availability of cellular phones equipped with a powerful processor and a built-in camera as an optical detector. An implementation example of a smartphone-based optical device is the prototype of a miniature analyzer of creatinine content in urine developed at the St. Petersburg State University (SPbSU) [16]. A review of such devices is given in [17]. In general, the attitude towards chemical analysis as an expert field is changing. We are observing preconditions for its wider and even mass use in the future.

In this situation, traditional sample-based lab spectroscopy no longer meets the current challenges. To bridge the gap, a new analytical methodology is required, including both new approaches to analysis (for example, specialization) and decision-making approaches, such as interim analysis or screening [1]. One of the answers to new analytical challenges is the development of optical multisensor systems. Such paradigmatic changes would require new technical solutions using the latest developments. One of the most striking examples of this kind is the growing analytical use of monochromatic light sources, such as lasers and light-emitting diodes (LEDs) to create multiwavelength sensors [18,19,20]. The appearance of pyroelectric detectors, such as Fabry–Pérot interferometer [21] and miniaturized spectrometers on their basis [2,3,4], stimulate the development of portable analyzers. Modern measurement interfaces and probes [22,23,24,25] as well as new light-guiding materials in a broad spectral range including mid-IR [26] enable flexible in-line spectroscopic monitoring of industrial processes. In general, the multisensor approach offers higher flexibility for the optical spectroscopic analysis. The variety of designs associated with the modern technical solutions enables the application of analysis on many samples and conditions, and allows one to carry it out at different distances from the object.

The recent progress achieved in the field of chemometrics for multivariate data analysis [27,28] is one of the key factors in the development of spectral analysis in general and multisensor systems in particular. The possibility of complete use of the redundant information supplied by modern instrumental methods leads to a paradigmatic shift in approaches to chemical analysis, which generally impacts the quantitative analysis, but can be used for the qualitative and spectral interpretation methods as well [29]. This is mainly related to the spectroscopic data, but there are other successful examples of the chemometrics application in chemistry [30,31].

### 2.2. Optical Multisensor Systems

The author’s work [5] systematically introduces the concept of optical multisensor system as a new class of analytical spectroscopic devices. In accordance with the proposed definition, OMS is a specialized spectrometric analyzer of low selectivity, including two or more optical chemical sensors or sensory channels (in the nomenclature of IUPAC [32]) optimized for a particular application.

The definition of multisensor system was initially introduced in electrochemistry for the gas analyzers called “electronic nose” [33] and was firmly established in the works on “electronic tongue” by Prof. Vlasov’s group at SPbSU [1,34]. The need to introduce a new term to an essentially spectrometric device is due to its conceptual differences from both traditional and modern process spectrometers. In addition, the OMS terminology reflects the new focus of optical spectroscopy towards the development of specialized analyzers as opposed to universal ones.

The main differences between OMS and traditional lab spectroscopy [6] are presented in Table 1. Being the embodiment of a new, i.e., multisensor, approach in optical spectral analysis, OMS has a number of features that allow it to be distinguished into a separate class of analytical devices. The initial specialization for a particular application that is embedded in the very design of OMS is its main conceptual difference from other spectrometers. There are also several other, less obvious differences of OMS.

OMS specialization consists in using the required minimum of constituting spectral channels on selected application-based wavelengths, instead of hundreds of channels in a spectrometer. Traditional spectrometric channels are evenly distributed over the entire technically accessible region to provide high resolution ability and versatility of analysis. The variables in high-resolution spectra are considered monochromatic (or resolved), i.e., taking a narrow wavelength interval that does not overlap with the neighbors. This is not always the case in practice, but the channel resolution from each other is highly desirable. On the contrary, OMS channels are generally wide; they can be spaced apart or strongly overlap, so that the very term “resolution” can even be inapplicable. High spectral resolution that is important for component identification is not critical in quantitative analysis. On the contrary, the possibility of using the most informative wavelengths and spectrum regions makes it possible to avoid areas of noise and irrelevant signals. Therefore, the resolution reduction has a positive effect of eliminating and averaging the data noise and hence improves the reproducibility and accuracy of the analysis. The use of chemometrics largely compensates for the loss of resolution during the transition from the laboratory analysis to multisensor technologies [35].

The human eye is a striking example of natural OMS. The variety of shades we perceive is the result of processing signals from three cross-sensitive optical sensors of low selectivity. Evolution has optimized the sensitivity spectra of individual eye sensors for orientation in the environment; therefore, color perception by humans and different species of animals do not coincide. In this terminological context, the optical multisensor system can be called “electronic eye”.

OMS selectivity to the analyzed substances is not required, but possible. It can be observed, if a pure component signal occurs on an individual wavelength, but this is not generally expected or pursued as a goal. For example, the use of LEDs, with their relatively high emission bandwidths that grow rapidly with the wavelength [36], intrinsically assumes the low selectivity of analyzer. Following the vocabulary of electrochemical sensors, one can talk about cross-sensitive channels [34]. In this case, the analysis automatically implies the use of chemometrics tools.

The measurement speed is particularly important for in-line analysis. Therefore, it is generally higher in process spectrometers than in laboratory ones. As a rule, the minimal spectrum acquisition time is several milliseconds, for example, in spectrometers with a diode-array detector (DAD) [37]. Avoiding the use of light dispersion in some OMS types and significant technical simplification of the device create the potential for increasing the speed of analysis by several orders of magnitudes, e.g., to several microseconds, which is demanded by some applications.

The problem of possible absence of the hardware standardization of OMS (which is embedded in the very design of lab spectrometers) is solved by using mathematical methods. Thus, the identity of analysis results for the same sample by two different OMS can be achieved using model transfer methods, despite possible differences in the measurements performed by non-standardized instruments [38,39,40].

Thus, the use of chemometrics becomes almost compulsory for building predictive models on OMS data. In widespread OMS used for a popular application, for example, in the analysis of common food products, equipping each device with its own (local) calibration model may be impractical. As an alternative, universal (also called global [41,42,43,44]) models can be constructed. The global model can be hosted “on the cloud” for its facilitated use by distributed sensor systems. For traditional spectrometers, it is typical that the software is located on a computer connected to the device. It includes a preinstalled chemometric model, which produces the analysis results, e.g., predicted concentrations. Optical multisensor systems, on the contrary, tend to have full autonomy, making use of a built-in microcomputer, which calculates and outputs the results.

A significant simplification of the design while maintaining the spectroscopic principle of data collection in the OMS leads to a manifold reduction in the analyzer cost and dimensions. Let us consider NIR spectrophotometers as an example. Its transformation into OMS can reduce its price hundreds of times, and its weight or dimensions, tens of times. OMS autonomy is also achieved by the rejection of a permanent connection to an external computer and by making the main power supply optional. The power required for the device operation can be supplied by a battery. The autonomy and diminutiveness of OMS determine their portability, which is one of the most useful properties creating new application areas for multisensor systems. The availability of OMS, together with their miniature size, allows the creation of distributed sensor networks using multiple devices simultaneously for complete control of a complex object. This can be done, for example, by installing the sensors in various points along the production line or the spatial coverage of territories for environmental monitoring.

High hardware adaptability is an important advantage of OMS. For technical adjustment of the optimal wavelengths to a new application, it is sufficient to replace the elements that determine the optical properties of the system channels, such as LEDs or filters. This creates opportunities for a modular OMS design, which paradoxically combines narrow specialization and versatility.

**Figure 1:**
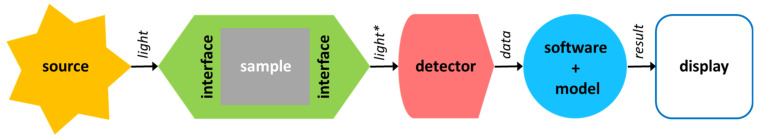
OMS information flow diagram (“*” denotes transformation).

A multisensor system can be thought of as a device for collecting, transmitting, and transforming information, i.e., as an information flow (Figure 1). The diagram equally describes the information flow of any spectroscopic technique; it is used here for the convenience of further discussion of OMS development approaches. The flow describes the source light transformation by the sample, its conversion to data at the detector, and then the modeling result using the software.

The primary information carrier is the electromagnetic radiation in optical spectral range emitted by a source (or multiple sources). Instead of conventional monochromators and gratings dispersing the light, the modern sources include optical devices producing monochromatic radiation, such as LEDs [18,20,45,46,47,48], lasers, or laser diodes [49], as well as optical filters [49,50,51]. The lighting can be stationary or can operate in a pulse mode [19].

The analytical information is collected by the interaction of light with the sample, which changes its spectral composition. The measurement interface is a set of optomechanical devices. It is responsible for the collection of relevant information during the light-to-sample interaction by transforming the light before and after the sample, as well as the sample shape at the moment of measurement. Examples of commonly used measurement interfaces are cuvette, flow cell, probe, and optical window. Analysis may occur at the contact between the sample and interface elements. Lenses, attenuated total reflection (ATR) crystals, mirrors, and optical filters are standard attributes of the measurement interfaces.

When passing through the detector, the information carrier changes. The light is converted into an electrical analog signal, which is then digitized. Consequently, the detector shapes an analytical signal of OMS information channels.

The final step in the OMS information flow is prediction, that is, the application of a preliminary built mathematical model to new measurement data in order to convert the analytical signal into a quantitative or qualitative result. For example, a calibration model is needed if the purpose of the analysis is to determine a sample component concentration. Thus, the predictive model is an indispensable element of the information flow that requires the use of an external or built-in computer. In addition to its device-controlling function, the computer serves to calculate and display the results of analysis. In some cases, as in the class discrimination tasks, the result can be displayed using color signals. For example, in spectroscopic detection of the tumor margin [52,53,54], green light usually designates “yes”, red—“no”, yellow—“margin”, and white—the measurement error.

An OMS classification has been proposed by the author [5]. OMS types are determined by their construction principles, i.e., by nodes of the information flow (Figure 1), where the OMS channels are formed to provide their specialization for an application. Thus, the channel formation can occur at the light source (OMS of the first type), for example, if monochromatic sources such as LEDs are used. It can also occur at the detector (type 2), e.g., when using (photo) diode arrays, or by selecting a few spectral intervals from high-resolution spectral data (type 3). The development examples for each OMS type will be shown later.

There are important special cases of OMS, in which the diagram in Figure 1 can be simplified. Thus, the right half of the flow after the sample can be replaced by human perception, i.e., by the eye as “detector” and the brain as “computer”. For example, the fluorescent color of a laser-illuminated sample indicates the presence of a certain chemical component, and the observed intensity indicates its concentration. Alternatively, the light source may be natural, such as sunlight. Moreover, the light source itself can be a sample, for example, in the spectral analysis of emitting objects—lamps, liquid crystal displays, etc.

### 2.3. Data Analysis in Spectroscopy and OMS Applications

The methods of chemometrics are used in many fields, but their main application is in analytical chemistry, primarily in spectroscopy [27]. Mathematical methods of spectral preprocessing [55,56,57,58,59], exploratory data analysis [55,60,61,62,63,64], variable and object selection [65,66,67,68], as well as their further improvement play a key role in OMS development success. There are several unsolved mathematical and methodological problems associated with the specific nature of OMS complicating their creation, data acquisition, and application. These include a small number of variables, their uneven distribution along the spectral axis, hardware standardization issues, and a potentially large number of measurements. This imposes certain restrictions on the use of common algorithms for spectroscopic data analysis and creates a need to develop new approaches.

In optical spectroscopy, there is a relationship between method selectivity and the importance of using multivariate data analysis. Thus, mid-IR and Raman spectra contain well-defined absorption bands, but due to their large number, the probability of overlapping peaks is high. Although the use of chemometrics is still optional in this case, it is recommended in most practical applications. The multivariate approach becomes critical for the accuracy of analysis based on NIR spectra, where “pure” variables are practically absent due to large bandwidths, as well as the possible influence of water absorption and scattering effects. A special case of very low selectivity is presented by the quantitative analysis of spectral data based on small shape differences of diffuse peaks and scattering profiles of the mixture components. An example of this kind is a developed method (Section 6.1) for fat and total protein determination in milk from visible and shortwave (SW) NIR spectra obtained in the transmittance [69,70,71,72,73] or reflectance mode [74].

One of the main issues related to OMS development is the mathematical optimization of the optical properties of OMS channels for a specific application including their working wavelengths, interval widths, and weighting functions. It was shown that in the case of complex mixtures, scattering media, and low-selectivity methods of analysis, the use of the traditional expert approach based on a priori knowledge of the spectral properties of analyzed samples may be suboptimal [70]. The same applies to well-known interval selection algorithms, such as interval partial least-squares (iPLS) [66,70] and genetic algorithm (GA) [65,73,75].

The methods of multivariate calibration and discriminant analysis as well as the corresponding chemometrics software, being well established in the analysis of traditional spectral data, may require some adaptation to the peculiarities of OMS themselves and to their new practical applications. Thus, the limited number of channels, typically a few instead of hundreds, prevents the use of spectral smoothing and derivative preprocessing [57,59], as well as some baseline correction [76] and variable normalization [55,56,58] methods on OMS data. In this situation, new approaches are necessary to increase the data information content in complex cases. The decrease in the number of channels may also require an increase in the number of samples in order to preserve the statistical representativeness of the data [77]. On the other hand, multiple linear regression (MLR), the classical least-squares approach shows in some cases better calibration results on a few preoptimized data variables than more sophisticated projection methods, such as partial least-squares (PLS) regression [40,78]. MLR simplicity presents a significant advantage in the analysis of big data, in particular, for autonomous process sensors having less powerful computers and limited data storage.

At the same time, the construction of accurate and reliable calibration models and their verification should be based on statistically representative, but possibly small and therefore carefully designed sets of samples. This and similar experimental issues have been considered by a separate scientific discipline called the design of experiment (DoE) [79,80,81] that will be more closely considered below.

The methods of multivariate curve resolution (MCR), also known as spectral unmixing [61,62,63,64,82,83], has important applications in various practical areas, including PAT [10]. The MRC method can be beneficial in the development of OMS, in particular, for extracting the spectra of pure components from the mixture spectra, e.g., in determining the optimal optical configuration of sensor channels. With OMS data, MCR is typically used for exploratory data analysis. However, for certain conditions, MCR can be used to construct multivariate calibration models on a few samples only, as shown in [84].

When developing and using OMS, special attention should be paid to the problems of model validation [55]. The need for constant comparison of analysis results obtained by different devices requires a reliable answer about the advantage of a particular method or technical solution. This is necessary to compare OMS with the full-spectrum method, as well as differing sensor types in a chosen application or different versions of the same OMS in the course of the development [53,54,70,74].

Model transfer methods can be used [38,39,40] to compensate for the lack of equipment standardization, i.e., possible measurement discrepancy by serial OMS of the same type. The intended manifold replication of OMS created for a single application and their possible worldwide distribution make universal (global) models [41,42,43,44] preferred to local ones, which need to be built for each device individually. The global modeling strategy is an important prerequisite for developing cloud-based data analysis solutions as an alternative to the modern desktop chemometrics software.

Inexpensive, compact, and fast OMS-analyzers are going to become the main drivers of PAT development. Process chemometrics covers a variety of topics and aims to answer many practical questions. What tasks are usually solved during the process analysis? What risks should be avoided or minimized [85]? How to find the necessary or optimal combination of sensors [86]? What is the best way to acquire spectral data and reference samples [87,88]? What methods of data preprocessing and analysis algorithms are better suited for a given analytical problem [89]? How to get an adequately accurate predictive model for process monitoring at minimal cost and effort [90]? How to make sure that data quality and model reliability are sufficient [91,92]? How to transform the acquired knowledge about the process into a control strategy [92]? These and other foundational questions should be asked and answered at the design stage of the process, before the data collection begins.

The literature review performed in [5] outlines the current state of science and technology in the development of optical multisensor systems and helps in paving the way for their further development. The importance has been shown of creating compact and inexpensive specialized devices based on spectroscopy, which significantly increase the efficiency of the analysis of complex objects in determining both individual components and cumulative chemical indicators. This expands the capabilities of express analysis, field measurements, and in-line monitoring of technological processes. The achievement of this goal requires solving a number of problems that form the scientific foundation of OMS development, starting with the general methodology for their creation.

## 3. OMS Development Methodology

### 3.1. General Approach to OMS Development

This section is devoted to the theory and practical aspects of developing multisensor systems. In the proposed approach, the OMS is intrinsically designed as a simplified alternative to the traditional spectroscopy (a full-spectrum method). The simplification is achieved through method specialization, which is based on a selection of the most suitable spectral intervals for analysis. The mathematical optimization is performed on the full-spectrum data of a designed experiment [5,45].

The OMS development cycle consists of five stages, including certain sequence of tasks [6], as shown in Table 2. Each stage serves to achieve a specific goal, and their results influence further development decisions. OMS design begins with an analysis of requirements of the intended practical application, which determine the properties and the budget of the developed analyzer. The study of method feasibility, its operability, and achievable accuracy, as well as the decision on the expedience of development itself, are made on the basis of preliminary measurements of model samples by the selected full-spectrum method or method combination.

The design stage ends with the optimization of OMS information channels carried out on the preliminary obtained data of the full-spectrum method. Each channel is characterized by the properties of the corresponding spectral interval: central wavelength, width, and weighting function; and possible channel configurations should be ranked by building and testing calibration models. Thus, finding the best set of intervals from full-spectrum data is a multiparametric optimization problem, the complexity of which grows rapidly with the number of channels.

### 3.2. Channel Optimization Algorithm

**Figure 2:**
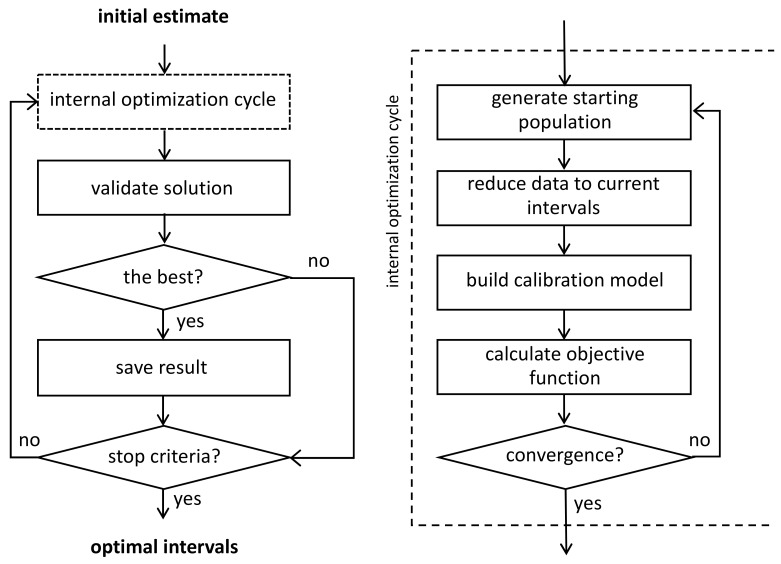
Block diagram of genetic algorithm for OMS channel optimization.

To speed up the calculations, an approach based on an adapted genetic algorithm (GA) [65] including two optimization cycles (Figure 2) has been proposed and tested in the work [73]. The outer loop performs a random change of populations (a set of parameters to be optimized) in order to increase the likelihood of finding the best or most acceptable solution. In the inner cycle, the so-called evolution of each population is carried out and a local optimal solution is found. At each evolution step, a multivariate calibration model is built for the currently tested intervals, and the root mean-square error (*RMSE*) of calibration (*RMSEC*) is used as the objective function that should be minimized. Each new internal optimization solution is validated in the external cycle, for example, using cross-validation (CV). The solution with the minimum *RMSECV* is saved. To reduce the calculation time, restrictions can be imposed on channel properties—their quantity, width, and relative positions to each other.

An important extension of the algorithm has been proposed, which allows including preprocessing parameters of the analyzed full-spectrum data, such as the derivative order, the smoothing degree, etc., in the optimization (together with the properties of the intervals). The proposed approach can be used to improve calibration models in the analysis of spectral data [5].

### 3.3. Illustration of OMS Development Stages Using a Practical Example

As an example, let us consider the development of LED OMS for the express analysis of milk based on the light scattering [69,70,71], as described in Section 6.1. The motivation for this study was the current demand for an inexpensive, compact, and portable analyzer for the determination of fat and total protein in milk. Based on the application requirements, the region of visible and short-wavelength NIR light (400–1100 nm) was selected for the analysis, test sample sets were developed [69,70], and full-spectrum measurements were carried out. The experiments have shown the efficiency of the method in the presence of a significant (exceeding natural) variation in the fat globule sizes. Using the calculated variable intervals, an optimal set of LEDs for constructing OMS was selected [73].

At the next stage of development, the measurement geometry and interface are optimized (Figure 1), as well as other variable technical parameters of the channels, such as radiation intensity and pulse frequency of the LEDs. In this case, the physicochemical properties of the sample should be taken into account: aggregation state, homogeneity, turbidity, flowability, optical density, shape (including the volume available for measurement), surface nature, etc. Optimization of the measurement technique is an expert task that requires participation of an analyst with knowledge of chemistry, physics, and spectroscopy, as well as data analysis methods. This work assumes the presence of a working analyzer, and it is carried out in the second stage, the purpose of which is to create an OMS prototype that meets the requirements. The initial prototype configuration is created based on the theory and mathematical models. Its further step-by-step improvement requires testing after each constructional change on a specially developed standard series of samples. The prototype refinement and testing loop continues until the specified requirements are met. It is important that the testing methodology allows model comparison of different prototypes with each other and with the results of full-spectrum analysis. In the milk example, spectroscopic determination of fat and protein can be performed in transmission [69,70,72] or diffuse reflection [71,74] mode. Tests of several prototypes and measurement geometries using the developed test set have shown the operability of both measurement modes and made it possible to determine the optimal path length.

At the prototyping stage, it is also necessary to ensure the required data quality, in particular, the measurement precision (reproducibility). The data quality is provided both technically and mathematically by normalizing the data with respect to a reference sample (the standard) in order to represent them in optical density units most suitable for quantitative analysis. In addition, the normalization minimizes the dependence of measurements on fluctuations in the light source intensity and on the instrumental drift. The choice of a reference sample is often a difficult task, for example, in in-line process monitoring. In this work, a reference-free analysis has been proposed [74] using the “internal normalization” approach (Section 6.1). It has been successfully tested for the determination of fat and protein in milk through a diffuse reflection probe.

At the third stage, the developed prototype turns into an analyzer that is ready for serial production. In the course of optimization, materials and constructional solutions can be replaced with elements, which are more suitable for production and operation, and to obtain a more reliable and economic design. At the second stage, the industrial model improvement consists in a cyclic testing of each new version using a test set of samples until it reaches the required quality and price. Thus, in the milk analyzer example, you will need to select the LEDs of appropriate quality and the probe materials regulated for food production.

To start working with OMS (fourth stage), it must be equipped with a predictive (calibration or classification) model. In the PAT practice, less accurate preliminary “fast” models are often used, which are then additionally trained on historical data in the course of analysis. A preliminary model can be obtained using a budget experimental design of the calibration set or by transferring a laboratory model to new conditions. The stage ends with the creation of a full-featured analytical device equipped with a working model that provides the required analysis accuracy.

The OMS data have their own specifics, which can influence the choice of the data preprocessing and calibration methods. Thus, the small number of variables and the expected (due to the channel optimization) absence of strong correlations between them create prerequisites for using the classical MLR regression [37] instead of the widespread modern projection algorithms of chemometrics, such as regression by the projection to latent structures, also called partial least-squares (PLS) method. However, MLR on preselected spectral variables gives excellent results. Its advantages are the simplicity and lower computation load, which are important for autonomous work of OMS. On the other hand, the small number of variables makes many data preprocessing methods, such as spectral smoothing, inapplicable. When analyzing time series, this limitation can be overcome by applying smoothing and other algorithms to variables in the time domain (Section 6.2) [6,57], in contrast to the traditional aspect, i.e., to the vectors of spectral data.

As is seen from Table 2, at the first four stages of OMS development, the versions of the analyzer are regularly compared with each other and with the full-spectrum method on a limited (as a rule) set of test samples. To increase the reliability of such a comparison, we have proposed a multilevel validation of calibration models by segmented CV (SCV) using segments of various sizes corresponding to different levels of the experimental hierarchy [5,19,70]. Such a hierarchy should be embedded into the DoE in order to check various factors. The factors can be, for example: the instrument precision (repeated measurements), the solid sample inhomogeneity (measurements at different points of the sample), the sample variability (different sources), etc. It has been shown that the comparative analysis of errors of different SCVs among themselves, as well as with *RMSEC* and independent test-set validation (TSV) *RMSEP*, allows a much better estimate of the model stability of future samples than individual validations. The multilevel (or nested) validation helps identify the shortcomings of the analytical method, and optimize the modeling parameters.

The multivariate model installed on OMS may need support in the course of its practical use. This is the content of the last fifth stage of development [6]. OMS model support is necessary to ensure that the required analysis accuracy is maintained. The support may include the following elements: self-testing of the spectral quality against an internal or external standard, testing new data for compliance with the model, taking control samples and their laboratory analysis followed by the assessment of prediction error tolerance, and continuous measurements in order to detect unwanted time deviations (instrument drift, noise growth, changes in the signal shape). If necessary, additional training or complete replacement of the model may be required.

The miniaturization and cost reduction of analysis incorporated in the very concept of multisensory approach (Table 1) open up opportunities for widespread replication of devices and their mass use in common applications, for example, for determining the nutritional value of milk. This creates the need, on the one hand, to provide many systems distributed around the world with an up-to-date model, and on the other hand, the ability to continuously update this universal (or global) model using data obtained by individual OMS working on-site. The possibility of building a global model and its transfer has been tested on the OMS for milk analysis considered here as an example. An important role in this example belongs to the same test set of samples that was developed above at the first stage.

The concept of global modeling assuming centralized work with data from distributed analyzers was implemented in the TPT-cloud software package [93,94] running on a remote server “on the cloud” at tptcloud.com (accessed on 15 May 2021) and available via a standard internet browser. The software provides mathematical support for the full cycle of creating and using OMS: optimize OMS channels, load data from various sources, conduct data preprocessing, build predictive models, and save them for practical use.

## 4. Designing a Calibration Experiment

### 4.1. General Construction Rules

The accuracy of analysis directly depends on the structure of data used to build and test the final or intermediate (widely used in the OMS development) predictive models. This section focuses on the design of the experiment for quantitative spectral analysis of mixtures. The construction of calibration models for several components on the same set of measurements is an important issue that has not been fully addressed by the theory [79].

One of the most important differences from the traditional DoE relates to a significantly larger number of levels (*l*) used for varying the values of calibration factors, e.g., component concentrations. A well-balanced calibration set should consist of a sufficiently large number of samples (*N*) uniformly distributed over the *k*-dimensional experimental space of factors. The main task, in this case, is to obtain the best model, i.e., to increase the accuracy of the subsequent prediction; thus, the optimal set of samples should be proposed before the experiment. Therefore, the way to a “good” calibration lies through reducing the risk of introducing various errors. This can be achieved by the following systematically formulated requirements given in paper [81].

Uncorrelated factors. Pairwise correlations between factors should be kept to a minimum. This requirement is of paramount importance. This is the only way to avoid indirect dependence, also called confounding, when the calibration model for one component is based on its correlation with another one and not with the measured analytical signal. 

Uniform filling. A well-balanced calibration set should be sufficiently representative and include many samples evenly distributed along the factor axes. Therefore, there is a need for a sufficiently large number of planning levels. The design matrix **C** (*N* × *k*) may include tens or hundreds of measurements depending on the analytical problem. 

Experimental space coverage. The regression model is intended for prediction on new samples lying inside a convex formed by training samples in the factor space. Otherwise, this is an extrapolation, and prediction reliability is not guaranteed. Therefore, one of the tasks of DoE is to provide maximum coverage of the experimental space with samples. The coverage percentage should be considered as the ratio of the volume of the convex *k*-dimensional polyhedron formed by the samples to the total volume of the experimental space. The filling is 100%, if all the vertices of the polyhedron contain samples. Emptiness of the border regions adjacent to the corners of the experimental space can have a particularly negative effect on the prediction quality in the presence of factor interaction, for example, due to a chemical complex formation between the components.

Built-in validation. A well-designed calibration experiment should include a predefined (a built-in) test set of samples or provide a way to create it afterwards. The built-in test set must be representative of the training set, and therefore, be generally similar to it. At the same time, it should not have close neighboring samples from the training set (the worst case is a coincidence). In addition, the test samples should not occupy the utmost positions of the coverage area at the boundaries of the experimental space. It is useful to have the ability, provided by the DoE scheme, to build a regression model both for the full set of samples and for the training set only, while the test samples are excluded. In that case, the exclusion of test samples should not lead to any imbalance and significant deterioration of the model. Despite the obvious importance of the problem, no DoE methods have been found in scientific literature that combine training and validation sets of calibration samples within one scheme. This shortcoming is inherent even in the classical comprehensive full-factor design that has samples on all levels of all factors.

Interpretability. The arrangement of samples in the experimental space is transferred to the factor space of the multivariate calibration model and can be subsequently analyzed, for example, in PLS score plots. The distortions underwent by the DoE scheme, when it is transferred to the space of latent variables (LVs) carry useful information about the factor significance in the model, about the presence of interfactor correlations and interactions, non-linearities and outliers, as well as about the physical meaning of LVs themselves.

Additional properties. Calibration DoEs built around a central point are well suited for the analysis of objects having an expectedly constant, e.g., natural, standard or target chemical composition. The sample of expected composition is then set into the center of experimental space and others are alternately added to its periphery. Such “centrifugal” schemes can be used to enrich an existing model with new samples and to expand the experimental space boundaries, which is not always possible in other DoEs. Some DoE methods (for example, random selection of samples) cannot be unambiguously described by a combination of *N*, *l*, *k* values. In order to reproduce the experiment in this case, it is necessary to store the entire design matrix of concentrations. Another important feature of any design is the building simplicity of schemes. The above-described additional properties of calibration DoE are not mandatory requirements, but they affect the method practicality and functionality.

### 4.2. Diagonal Design

Based on the formulated requirements, a new approach to the experimental design of a multicomponent calibration experiment has been proposed. In accordance with this approach, called *diagonal design* (DD), the samples are systematically distributed along the diagonals of the experimental space represented by a square, cube, or hypercube. Consequently, the mutual correlations of the component are minimized, and the uniformity of filling the factor space is ensured. DD is unique in that it has a built-in test set. Due to its simplicity, it can be implemented without a computer.

The main assumption of DD is the linear dependence of the analytical signal on the concentration. However, some moderate deviation from linearity is not an obstacle for the practical application of the method. The factors in DD must be controlled so that the values of analyzed concentration can be arbitrarily varied. Multivariate calibration design follows three simple rules:

(a) The experimental space is a hypercube: the intervals of all factors are split into the same number of levels;
(b) The samples are uniformly distributed along the diagonals of the hypercube;
(c) Each level of each factor is represented by one and only one sample (Latin hypercube condition).

Two additional rules apply to the test set:

(d) Test samples should not occupy adjacent levels of any dimension of the experimental space;
(e) Edge samples on the diagonals are not used.

The most practically important case is the simultaneous calibration for two components with or without a central point. The central sample, if it exists (its absence does not affect further filling) is always placed in the origin and gets ID = 0.

**Figure 3:**
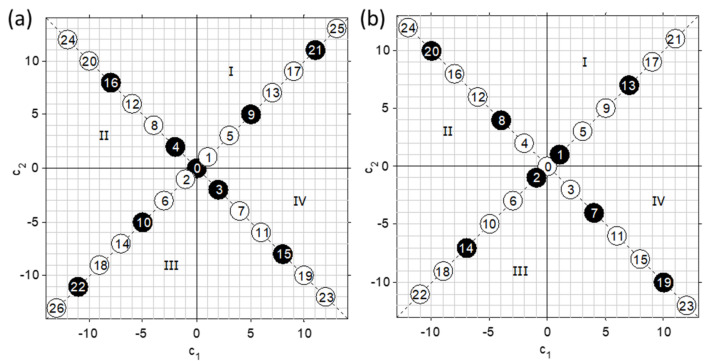
Two-factor diagonal schemes with the central point for: (**a**) 27 experiments with a test set (black circles) starting with the sample ID = 0 ($d2_{0v0}^{27}$); and (**b**) 25 experiments with a test set (black circles) starting with the sample ID = 1 ($d2_{0v1}^{25}$); Roman numerals designate the quadrants.

The ID number of subsequently added samples increases incrementally (Figure 3). The samples are added from the center to the periphery following the “rule of the cross”: the first sample is placed at the nearest diagonal position of the first quadrant, and subsequent odd elements alternate between semi-diagonals in quadrants I and IV, always taking the next vacant place. After adding an odd sample, its even “twin” is simultaneously placed in a symmetrical position on the corresponding adjacent semi-diagonal in quadrant II or III. The advantage of the proposed diagonal scheme is its practicality. A two-component experiment is easy to design on checkered paper. The conditional coordinates used here are convenient for the creation of a scheme. After that, they can be easily recalculated, e.g., into concentrations.

**Figure 4:**
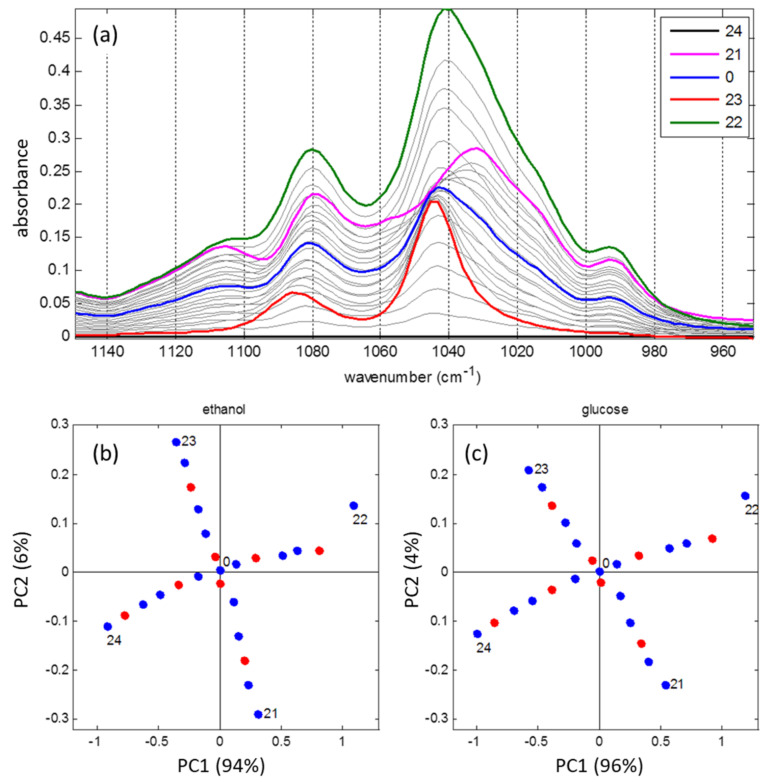
Application of a diagonal design $d2_{0v1}^{25}$ (from Figure, where c_1_ is a decreasing concentration of glucose and c_2_ is an increasing concentration of ethanol) for the determination of ethanol and glucose in their aqueous mixture by IR-spectra: (**a**) spectra; and PLS score plots (**b**) of ethanol and (**c**) of glucose in the first two latent variables.

Two-component schemes and their elements have been tested in the works of the author and other researchers. Figure 4 shows the results of simultaneous calibration for ethanol and glucose from IR-spectra of aqueous solutions of the components mixed according to the scheme $d2_{0v1}^{25}$ (Figure 3b). (Designation $dk_{DSvVS}^{N}$ of an experimental design has been introduced; d means diagonal design; *k* is the number of factors; *N* is the number of samples; *DS* and *VS* are the sequence numbers of the first samples in the training and validation (if any) sets, respectively.) This test series of samples was used to compare the full-spectrum method (Figure 4a) and prototypes of the developed OMS for the fermentation process monitoring [32]. It has been shown that the presence of a built-in test set of samples in addition to full leave-one-out cross-validation (LOOCV) on the entire set serves well both for determining the number of latent variables (LVs) in the PLS regression and for comparing models or analytical methods. Cross-arrangement of samples can be easily traced on the graph of PC-scores (Figure 4b,c), thus facilitating their interpretation. In particular, it reveals the presence of an outlier, the presence of correlation of spectral signals, and possible non-linearity of the response.

### 4.3. Diagonal Design Generalization for Three and More Components

**Figure 5:**
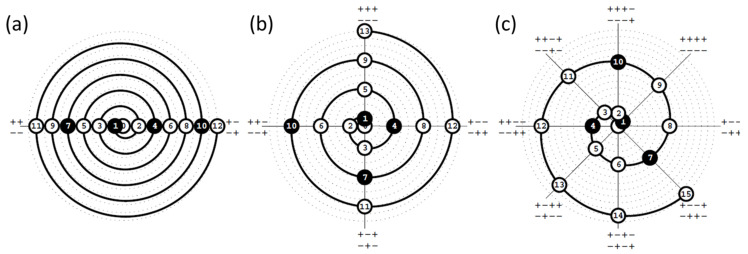
DD filling scheme with four pairs of test samples (black circles) for (**a**) two, (**b**) three, and (**c**) four factors.

The above-described algorithm for alternating filling of diagonals, starting from the center, can be generalized for experimental space of any dimensionality *k* [81]. Its clear geometrical illustration is the Archimedean spiral in polar coordinates (Figure 5), which helps in planning calibration experiments without a computer.

**Figure 6:**
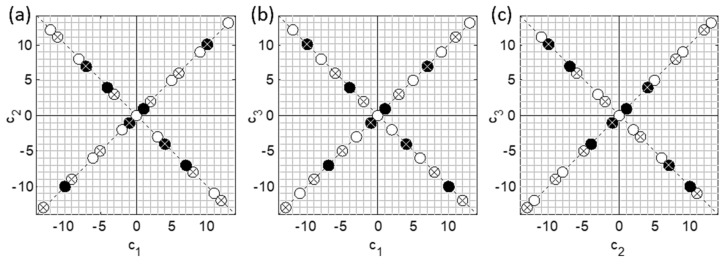
Diagonal scheme for three factors with 27 samples, with a central sample and a test subset (black circles), starting with sample #1 ($d3_{0v1}^{27}$). In each two-factor projection (**a**–**c**), the samples with negative values of the third (hidden) factor are marked with a cross.

To illustrate the DD filling algorithm, let us consider a three-component system in Figure 5b. Each axis outgoing from the origin represents one of the four (2*^k^*^−1^) diagonals of the cubic experimental space. The algorithm operates on semi-diagonals identified by unit direction vectors pointing to the respective vertices. The direction vectors are designated by “+” and “−” signs at the end of an axis in Figure 5. For example, $\frac{+-+}{-+-}$ in Figure 5b denotes two halves of the diagonal connecting the cube vertices indicated by the vectors (1,−1,1) и (−1,1,−1). The central sample #0 is set at the origin. Each subsequent enumerated position along the developing spiral (circles in Figure 5) determines the diagonal being filled, and hence the centrally symmetric pair of experiments located on it. The conditional coordinates of these experiments are obtained by multiplying the ordinal number of the current spiral positions by two corresponding opposite direction vectors. Thus, the central element gets the coordinates (0,0,0), moving to the position 1 adds points (1,1,1) and (−1,−1,−1), the position 2 generates (−2,2,−2) and (2,−2,2), etc. The resulting three-dimensional DD scheme for 27 samples is presented in Figure 6 using pairwise projection planes.

The spiral scheme also offers a simple rule of thumb for selecting the test subset samples. The first pair of test samples follows from the design scheme itself (position 1 in Figure 5b). Then, every third position along the developing spiral forms a pair of test samples that meet the above requirement (d).

Two- and three-component calibrations are the most important in practice. Further generalization of DD for four and more factors is complicated by the loss of filling invariance. In other words, different sequences of diagonals in the algorithm lead to different schemes. Therefore, the paper proposes an algorithm for finding an optimal sequence of diagonals in the filling scheme. To create effective calibration DoEs for four and more components, several extensions of DD have been proposed, in particular, those based on the rejection of the Latin hypercube.

The theoretical principles of constructing training and test sets for multicomponent calibration, as well as the developed DD schemes, were successfully applied in the OMS development for a number of applications, in particular, for determining the total fat and protein in milk and in-line monitoring of various processes in PAT (Section 5). The reader can design the required diagonal calibration scheme for any number of components using Matlab code published in [79,81].

## 5. Optical Multisensor Systems in Process Analytical Technology

### 5.1. The Concept of Project Trajectory

Process analytical technology is an important branch of modern analytical chemistry. Research in this scientific field covers a wide range of processes—from the lab synthesis kinetics to industrial production plants and environmental monitoring. Today, PAT is rapidly developing, thus giving momentum to research in the field of spectroscopic analysis and multivariate data modeling. This section is devoted to the theory of development and application of OMS in PAT. A methodological concept of the process *trajectory* in the space of analytical variables has been proposed and systematically elaborated [10]. Recommendations are given on its use in process monitoring, control, and optimization. In particular, a methodology of the development cycle of spectroscopic methods for process analysis has been formulated, and the methods of solving its main tasks have been systematized.

**Figure 7:**
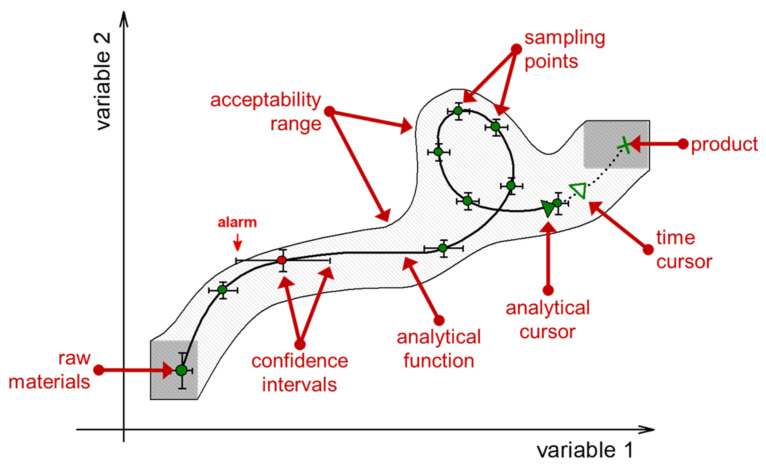
Process trajectory schematic: the progressed (solid line) and the predicted (dotted line) trajectory, sampling points with an error estimate (green circles), analytical (green triangle) and time (hollow triangle) cursors, the area of normal process course (shaded), areas of acceptable quality of row material and product (gray rectangles).

The trajectory exists in the space of variables chosen by the analyst for presenting the process course. In this sense, the trajectory is a projection of the whole process as a complex phenomenon onto the selected space. One can select an infinite number of possible spaces and the resulting trajectories of the same process can differ strikingly. The set of required variables forming the analytical space is determined depending on the presented analytical problem. The analytical space is schematically represented in Figure 7. In the following presentation, this diagram will be used to illustrate and discuss the main properties of trajectory.

**Figure 8:**
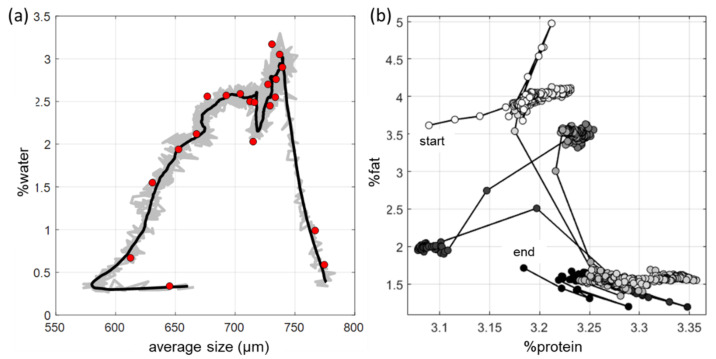
Examples of two-dimensional trajectories using in-line prediction: (**a**) pellet coating process; original (gray line) and smoothed (black line) trajectories, as well as reference measurements (circles); and (**b**) milk production process in mass fraction coordinates of fat and total protein; the gray color gradient indicates process time.

Being a natural attribute of all process types, the multivariate trajectory is a convenient tool for systematically considering various PAT aspects. Any tasks of analytical process control can be considered from the point of view of capturing, transforming, and subsequent use of the respective trajectories. Practical examples of project trajectories [10,95] are shown in Figure 8.

### 5.2. Process Analysis Workflow

**Figure 9:**
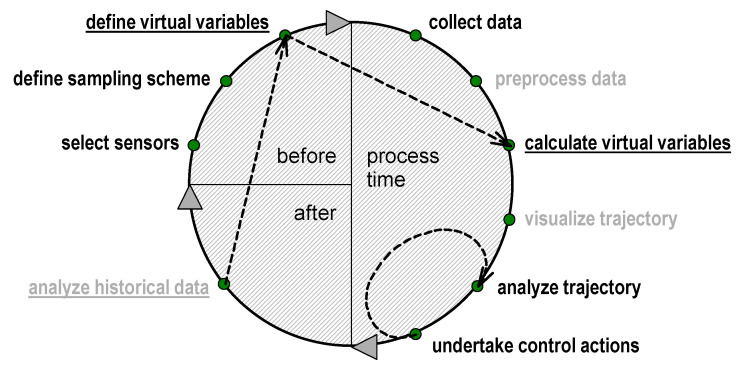
Generalized scheme of process analysis: optional actions are marked in gray; underlined font indicates application of chemometrics.

The process analysis is represented by a cyclic diagram in Figure 9 [10]. This diagram is divided into three sectors, containing the actions before, during, and after the processing time (before the next cycle). Cyclicity is an important concept that reflects the need for effective use of the accumulated (in the form of data and models) experience. The scheme is designed to assist the development of PAT methods using OMS. The data accumulation is preceded by the choice of optical sensor channels—physical variables of the analytical space, in which the primary acquisition of the process data is performed. The main requirement for the sensor channels is their ability to deliver the data necessary for solving the problem at hand. The starting set of sensor channels can be redundant. A process spectrometer is often used as the first prototype in the OMS development. The measurement mode and interface, reference sample, and data acquisition conditions are selected according to the task (Section 3). With repeated passes of the cycle in Figure 9, the OMS design and the data acquisition parameters can be improved based on the collected experience in order to achieve the optimal (from the application point of view) configuration of the system channels.

The application of chemometrics makes it possible to use virtual variables resulting from the prediction by a calibration (as well as discrimination or another) model built on already collected data. As a rule, this stage is accompanied by compression of the analytical space using regression and variable selection [45,63,70,73] methods. 

The raw data collected during the process can be preprocessed and help visualize the trajectory using a previously created model. Almost any projection method—PCA, PLS regression, or MCR [61,62,63,64] can be used to resolve and render trajectory from the multivariate spectroscopic data. Trajectory visualization is needed, first of all, to perform the process monitoring. Progressing trajectory observed in the course of the technological process is subject to continuous analysis, i.e., visual assessment by the operator or automatic interpretation using software, for the timely detection of an “alarm” situation. This situation means that the process is out of the normal course (Figure 7) and requires any control actions for its elimination. The cycle “impact on the process—trajectory analysis” is repeated until the problem is completely eliminated (loop in Figure 9).

The data obtained after the process completion become historical and can be used to create a new model or update a previously created one. The historical data can also be used for exploratory analysis for in-depth understanding and eventually for the improvement of analytical methods and even the manufacturing process itself. Before the beginning of each new analysis cycle, OMS can be improved both technically by means of further optimization of the channel configuration and in terms of the analytical space, by choosing new physical and virtual variables. The effectiveness of the improvement should be thoroughly tested using a standard validation set of samples and appropriate approaches such as nested validation (Section 3).

### 5.3. Trajectory Application in Process Analytical Technology

The concept of trajectory is very useful when discussing process analysis in PAT. The following PAT goals are usually distinguished: process monitoring, control, understanding, and optimization. Each of these goals includes a number of specific practical tasks that can be solved using OMS. Table 3 describes the goals, objectives, and solutions in terms of analytical space and multivariate trajectory [6,10].

Modern optical analyzers—process spectrometers and OMS—are the most important PAT tools. Their development and implementation into production is a key condition for solving the main task of process analysis, which is continuous monitoring of processes in real time. The number of monitoring objects is constantly growing, which brings process control to a qualitatively new level. Strengthening analytical control in manufacturing through the widespread use of in-line measurements and, as a result, a deeper understanding of technological processes contributes to higher product quality.

## 6. OMS Development and Application Examples

This section contains illustrative examples of the development of OMS and corresponding new or improved analytical techniques in various areas of practical application: food and pharmaceutical industries, biotechnology, medical diagnostics, and ecology. The presented systems are at different stages of development (Table 2) that follow the principle of simplifying the full-spectrum method by optimizing it for a selected application. The optimization is based on preliminary measurements using a laboratory or process spectrometer.

### 6.1. Scatter-Based Determination of Fat and Protein in Milk

A new optical method for the analysis of milk for fat and total protein content using spectrally observed light scattering in the 400–1100 nm region has been suggested [69,70]. It was presented as the basis for the development of an OMS on light-emitting diodes (LEDs).

**Figure 10:**
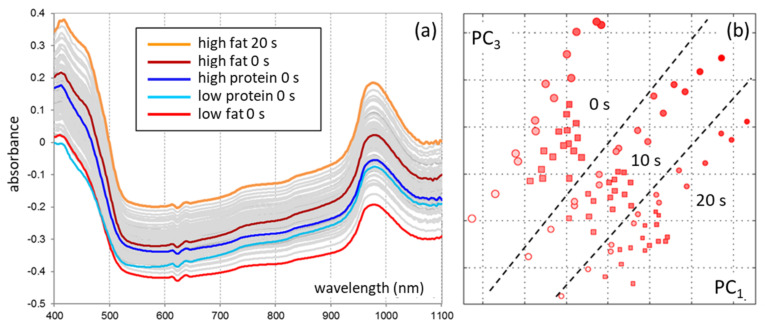
(**a**) Spectra of 96 measurements of the calibration samples (gray lines) with highlighted characteristic samples at different homogenization times; and (**b**) their representation in the PC1–PC3 plot of the PLS model for fat content. The color saturation corresponds to the fat concentration and the marker size corresponds to the sample homogenization degree; circles represent samples with predominant variation in fat, and squares—in protein content.

A prerequisite for the accurate quantitative determination of colloidal components of natural (non-homogenized) milk is the ability of the spectral method in combination with chemometric data analysis to distinguish low-selective spectral profiles of scattering particles having different types and sizes: protein micelles (mainly 80–200 nm) and fat globules (1–15 μm) [71]. To validate the method, a series of calibration experiments was performed with artificial milk samples prepared according to the principles outlined in Section 4, including the use of DD [69,70]. To account for natural variation in the fat globule sizes, each sample was analyzed both in its initial state and after a step-wise ultrasonic homogenization. Analysis of full-spectrum data (Figure 10a) of the laboratory prototype and exploratory factor analysis of a PCA model have shown the feasibility of accurate determination of fat and total protein (Table 4) in natural milk in the presence of particle size variability. The sample grouping in accordance with their homogenization degrees confirms the result, as observed in Figure 10b [70].

Global calibration models were built for the initial full-spectrum method implemented on the basis of a laboratory diode-array spectrometer [43]. Both models were based on more than 1000 samples collected during a year in the district of Samara and adjacent regions. *RMSE* of both fat and protein determination were about 0.1% in the fat and protein content ranges of 1.55–4.97% and 2.27–4.25%, respectively. The possibility of model transfer to another spectrometer of the same type by the slope-and-bias correction method has been shown. The model is transferred without any significant loss of accuracy. The first OMS prototype was built on the basis of three LEDs in the visible region, alternately illuminating milk samples placed in a Petri dish through a light guide connected from below [72]. Detection was carried out using a digital camera photographing diffuse light spots on the sample surface. Calibration models based on the selected image features have shown the fundamental feasibility of LED-based OMS analysis for the determination of fat and total protein in natural milk.

LED configurations for OMS for the simultaneous determination of fat and protein in transmittance mode were calculated using the optical channel optimization algorithm on full-spectrum data (Section 3). The system with 7 LEDs at selected wavelengths proved to be almost as accurate as the original method [70].

**Figure 11:**
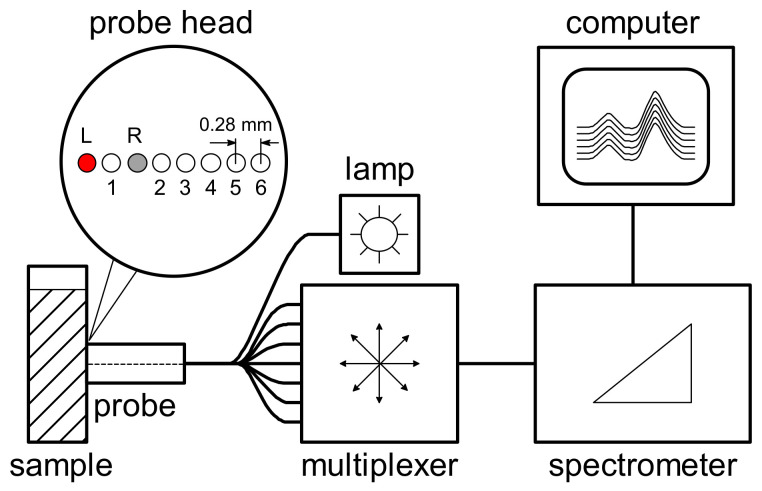
Experimental set for the space resolved spectroscopic measurement. The arrangement of channel on the working surface of the probe is indicated by numbers 1 to 6; L and R are illumination and comparison channels, respectively.

A separate full-spectrum experiment (Figure 11) has shown the effectiveness of using spatial resolution (in addition to the spectral information) to increase the accuracy of milk analysis in diffuse reflectance mode [74]. The spectral detection at different distances from the light source was carried out using a probe with an array of eight fiber channels. For the data analysis, we proposed a reference-free method, where one of the probe channels was used for internal comparison.

**Figure 12:**
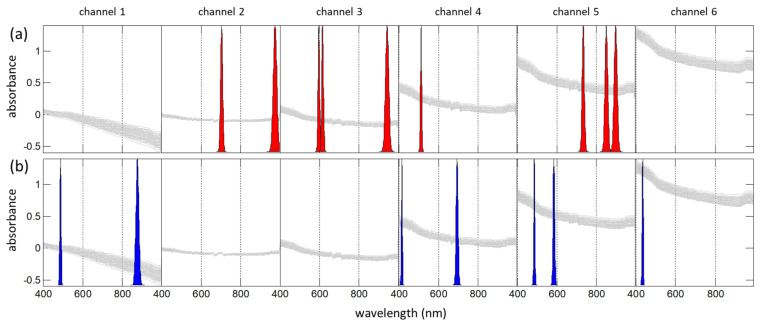
Calculated configuration of LED-OMS with spatial resolution for the analysis of milk: (**a**) fat content and (**b**) total protein content; LED emission peaks of the corresponding OMS are shown over the original spectra (gray lines).

The calculation of the LED-OMS was carried out on augmented spatially resolved data, where all channel data of the same measurement are connected into a single spectrum (Figure 12). Each spatial channel has its own optimal set of LED sources (the invention is patented [48]). Being easy to maintain, diffuse reflectance OMS for milk analysis are well suited for the field and in-line analysis. The need to use light guides means weaker signal intensities than in a transmission measurement. Nevertheless, the method has shown almost the same accuracy (Table 4). This can be explained by the positive effect of spatial resolution in combination with the channel optimization and reference-free measurement improving the precision.

### 6.2. In-Line Quality Monitoring of Solid Pharmaceutical Forms

A number of practical OMS development examples are presented for the in-line quality monitoring of solid pharmaceutical forms, namely: residual moisture content [57,95], concentration of an active pharmaceutical ingredient (API) [10,95], layer thickness of a polymer protective coating [95], counterfeits detection [96], and real-time prognosis of expected product solubility [97]. A DAD process spectrometer in the NIR region was used as the main full-spectrum method. The analyzer was equipped with a specially designed diffuse reflection probe immersed in the process medium [22].

**Figure 13:**
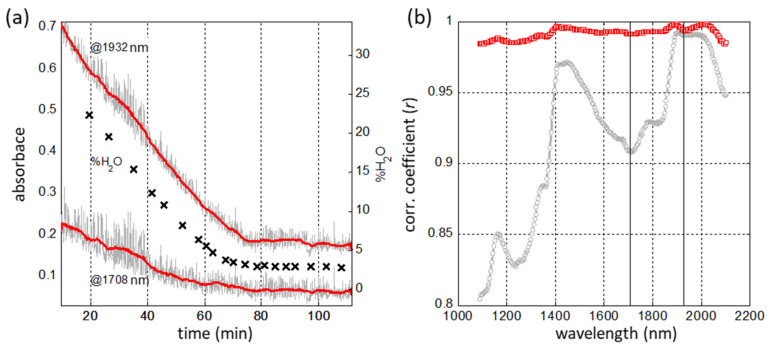
In-line determination of residual moisture during a granulate drying process: (**a**) time dependences of the row (gray line) and smoothed (red line) spectral intensities at selected wavelengths, as well as reference values of %H_2_O (crosses); and (**b**) the predicted versus measured plot of the PLS regression on smoothed data; the calibration and test sample sets are designated by the hollow and solid markers, respectively. Time smoothing of the data was performed using the moving average with a window width of (**a**) 47 and (**b**) 15 points.

One of the issues of in-line analysis of pharmaceutical powders and granules is the lack of water determination accuracy due to the high turbulence of fluidized-bed drying processes, which leads to strong variations in the total spectral intensity. It has been shown [57] that the mathematical preprocessing of spectra (the so-called scatter correction), which is traditionally used to eliminate this effect, simultaneously eliminates useful spectral information associated with the dependence of light scattering on the moisture content of the powder. This dependence is manifested in the fact that even the spectral wavelengths that do not belong to the absorption bands of water have a high correlation with the mass fraction of water in the granulate (Figure 13a).

To obtain the maximum accuracy of water determination, the modeling was performed on a representative set of designed experimental data (25 process batches, 16,303 in-line spectra, 301 reference samples). To preserve the entire data variance correlating with the water content, the stochastic fluctuations in spectral intensities were eliminated by smoothing along the time axis using the moving average method. Such preprocessing can significantly improve the data quality, and helps to avoid the information loss caused by the scatter correction. The use of DoE in combination with the time-wise smoothing made it possible to reduce the *RMSE* of %H_2_O (g/100 g) content prediction from the usual 0.3% to 0.1% in a wide concentration range (Figure 13b) [57].

**Figure 14:**
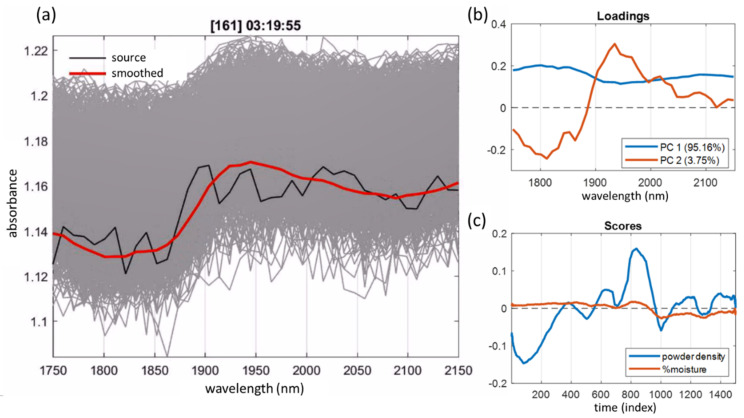
(**a**) Process spectra: all data (gray lines), raw spectrum with index 161 (black) and spectrum 161 after the data smoothing in the time domain using the moving averaging method with the window width of 101 points (red line); (**b**) PCA loadings and (**c**) PCA scores for smoothed data.

Another example illustrating the effectiveness of time-domain smoothing is the data analysis of a low-resolution OMS working in a narrow spectral region. The system was installed in the powdered food additive production line for the monitoring of moisture content (the composition cannot be disclosed because of the manufacturer requirement) [6]. The data are strongly affected by noise, which cannot be eliminated by spectral smoothing due to the insufficient number of variables (Figure 14a). Calibration is complicated by the product inhomogeneity and density variations in the flow. PCA exploration analysis did not reveal any useful internal structure in the raw data. However, after the variable smoothing in the time domain, the PCA loadings showed spectral features characteristic of water absorption (PC2 in Figure 14b), which indicates the feasibility of its quantitative determination. The respective PCA scores reflect the project trajectory (Figure 14c) in this case. PC1 most likely reflects the observed decrease in the spectral background, which responds to the powder flux density. In the given example, the chosen data preprocessing played a decisive role in substantiating the method feasibility.

Another process that requires in-line monitoring is the protective coating of pellets with a polymer shell, which provides a sustained release of the API in the patient’s digestive tract (the so-called targeted delivery). In the first series of experiments, the expediency of combining in-line methods of NIR and Raman spectroscopy was shown to obtain an informative process trajectory, including the main quality factors of the produced pellets: the quantities (thicknesses of the corresponding coating layers) of the applied API and the protective coating, as well as the humidity regime were maintained during their spraying. The combined data of the methods are well suited for exploratory analysis, in particular for MCR, which provides an in-depth understanding of the process. The PLS calibration model for water content built on the combined data gives a higher prediction accuracy than either method alone. Such synergy is absent for determining the polymer coating thickness, where NIR spectroscopy showed the best result.

A separate task in the analysis of pellet manufacturing processes and in the development of the corresponding OMS is the profile prediction of the API release from pellets based on the process NIR (the concept of feed-forward control, Table 3). The dissolution kinetics determines the quality of the produced pellets, and its in-line monitoring is necessary for the real-time product release (RTR), which is an important task of the PAT. The designed experiment [97] included 12 process batches with two types of the coating material and different sets of conditions. Solubility tests of the pellet samples taken at different production stages from the process environment showed that the fraction of released API φ versus time *t*, regardless of the material and coating thickness, is adequately described by an equation with two constants *m* and *k*, similar to the kinetics of autocatalysis, where *m* is responsible for the release rate (slope), and *k* reflects the induction period (delay):

$$\varphi \left(t,m,k\right)=100k\frac{exp\left[\left(m+k\right)t\right]-1}{m+kexp\left[\left(m+k\right)t\right]}$$

**Figure 15:**
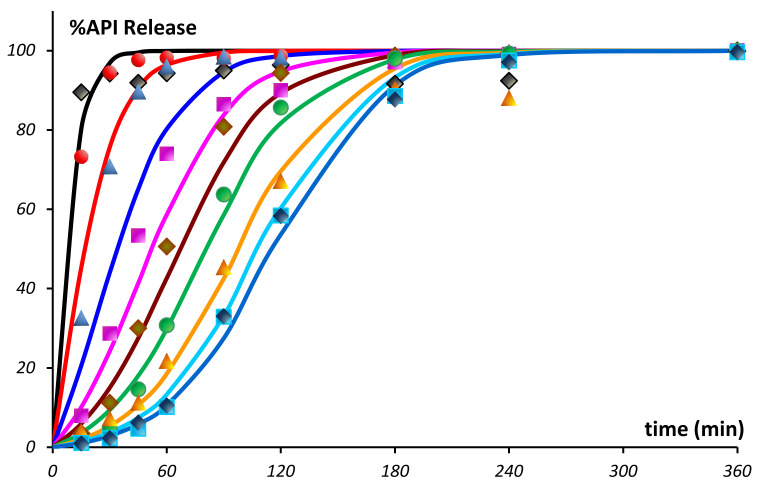
Sample dissolution test data (markers) and in-line prediction (lines) of API release for a test batch of the pellet coating process.

The study has shown that the parameter *m* depends on the coating material only, and thus, it can be calculated from the respective batch data using the method of successive Bayesian estimation, according to which the data of individual batches are processed one by one, and the estimates obtained by a non-linear approximation of the previous batch are used as a priori information for the next one. Another constant *k* is closely related to the applied coating thickness (spectrally observed through the total mass of the deposited material) and, therefore, can be determined using a calibration on the process NIR spectra. A validation using individual batches (Figure 15) has proven that the method is effective for practical in-line prediction of solubility profiles, and the obtained results provide valuable information for further OMS development and RTR implementation [97].

In a separate experiment, the feasibility of determining the pellet coating thickness through their average size was shown by analyzing digital images of samples taken from the process environment [98]. The approach can be presented as the basis of an OMS for express analysis at-line or can be modified into an in-line method.

### 6.3. Process Monitoring in Biotechnology

The third example illustrates the development of different OMS for the monitoring of biotechnological *S. cerevisiae* yeast fermentation process based on IR, NIR [35], and fluorescence [49] spectroscopy. A multisensor IR analyzer determining the concentrations of ethanol, glucose, and fructose using in-line measurements in the attenuated total reflection (ATR) mode was developed. Two types of ATR probes based on polycrystalline infrared fiber (PIR) with different ATR elements were used: diamond crystal (DATR) and an inexpensive interchangeable PIR loop (LATR). Two spectrometric technologies were also tested in developed OMS: a diffraction-grid spectrometer with a pyrodetector PYREOS (GrS) and a tunable interferometer of the Fabry–Pérot type (FPI). The spectral “fingerprints” region of 1150–950 cm^−1^ was chosen, which contains distinct absorption bands of the studied components [35].

The development approach described in Section 2 was used, which is based on simplifying the FTIR-DATR “gold-standard” method by means of replacing the spectrometer or the probe. Three OMS prototypes were developed and characterized using the test sample sets of 25 binary ethanol–glucose (EG) and fructose–glucose (FG) mixtures mixed according to the $d2_{0v1}^{25}$ scheme of DD (Figure 3b) [81]. The IR spectra of the full-spectrum method for the ethanol–glucose system are shown in Figure 4a. The transition from FTIR-spectrometer to GrS or FPI, as well as the replacement of a diamond probe with an inexpensive and easily replicable LATR are associated with a loss of spectral resolution and signal quality. Nevertheless, the obtained multivariate models retain high accuracy (Table 5), which is practically acceptable for a number of process monitoring applications, for example, in the food industry. The performance of the GrS-LATR system has been confirmed by tests in a fermentation process environment in a laboratory bioreactor.

In the OMS development, therefore, the chemometric data analysis largely compensates for the lack of selectivity. The considered results show the fundamental possibility of creating optical multisensor systems in the IR region for the analysis in the biotechnological process environment, which determine the content of ethanol, glucose, and fructose in concentrations that are typical for fermentation. The ability to determine various carbohydrates independently gives an undoubted advantage over existing single-channel, for example, refractometric analyzers.

Two OMS prototypes for in-line monitoring of *S. cerevisiae* fermentation were developed on the basis of fluorescence spectroscopy. One of the problems of both simple and two-dimensional (2D) fluorometric analysis of such media is the strong superposition of weak signals of biological fluorophores on the excitation band [49]. It is especially challenging when the fluorescence signal is weak and appears against a noisy background. The emphasis of the study was placed on overcoming these effects using the new methods of data acquisition and their multivariate analysis.

The first example shows a positive effect of an additional NIR radiation source from a single-channel light-scattering biomass sensor that was present in the bioreactor. The additional band with a maximum at 870 nm in the observed in-line spectra significantly improves the accuracy of biomass prediction compared to using the individual regions of fluorescence or NIR sensor separately. An explanation was found using MCR, which showed that the scatter intensity dependence on the yeast cell concentration makes it possible to distinguish the increasing fluorescence of intracellular fluorophores from the decreasing fluorescence of similar substances in the fermentation medium.

To develop an in-line monitoring method for *S. cerevisiae* cultivation by means of two-dimensional fluorimetry, 39 excitation-emission spectral matrices (EEM) including fluorescence spectra at 24 excitation wavelengths were collected during the process. Due to the strong dominance of the excitation band in the spectra, the initial process data were poorly suited for traditional factor analysis; any standard preprocessing was found to be destructive for weak fluorescence signals. A new MCR-based algorithm has been developed for the analysis of this data, which allowed resolving both the process trajectory and pure two-dimensional EEMs of individual fluorophores [49].

The tests of various optical spectral methods in four fermentation processes of the yeast *S. cerevisiae* have shown that OMS development is promising, including systems based on both IR spectroscopy and fluorimetry (one- or two-dimensional). The maximum use of spectral information, as well as the use of exploratory data analysis at the method development stage, is important for the fermentation process monitoring.

IR systems on the basis of modern detectors with the help of chemometrics are able to selectively and accurately determine ethanol and various carbohydrates. Fluorimetry can determine the biomass content and oxygenation of the medium. Therefore, the methods can be successfully combined to build informative analytical spaces and process trajectories (Section 4) [10].

### 6.4. Medical Diagnostics

**Figure 16:**
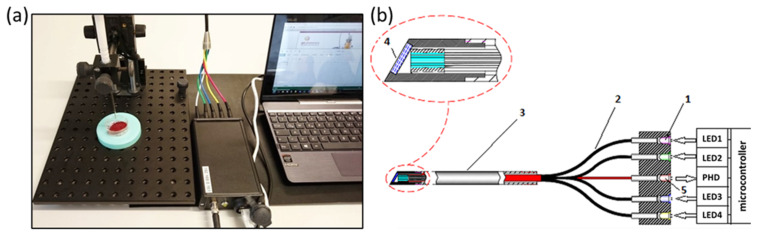
(**a**) Experimental setup and (**b**) construction scheme of the improved prototype B of the LED OMS: 1—LEDs, 2—fiber optic cables, 3—stainless steel tube, 4—sapphire window (the prototype A of the probe had a rectangular shape and did not have a protective window).

Medical diagnostics is one more practically important area for the application of OMS. In the present series of studies, several prototypes of optical diagnostic analyzers for the tumor border detection of the human kidney cancer were developed for the first time [19,46,52,53,54,99]. Spectral histopathology is a relatively new approach in oncological diagnostics, which can reduce the probability of error during surgery. In the first example, two prototypes of LED-OMS based on NIR spectroscopy, called A and B, were developed and tested (Figure 16) [19,46]. The system operation was based on a rapid alternating illumination of the sample at several wavelengths and measuring the diffusely reflected light by a photodiode detector (PHD). Four LEDs were chosen for the analysis with central wavelengths near the absorption maxima of water (0.94 and 1.44 μm) and lipids (1.17 μm), which are known markers of some cancer types. An LED with the maximum emission at 1.30 µm was added to correct the light scatter effect. Hence, it served as an internal reference channel in accordance with the reference-free measurement concept (Section 3). A preliminary analysis on the full-spectrum NIR data justified this choice of channels [46]. The aim of the study was to experimentally confirm the fundamental possibility of recognizing the pathologically altered biological tissue by the proposed method on a limited number of available clinical samples. The tumor can be detected through the quantification of spectrally active chemical components, i.e., water, lipids, glycogen, and other cancer markers, as well as the morphological properties of tissue.

To increase the reliability of the results, the experimental design had a few hierarchical levels, including repeated measurements at several sample points [5,46]. Thus, the resulting measurements reflected the tissue variability both within a sample and between them. Discriminant analysis (DA) of the data was carried out by the PLS-DA method, based on the construction of a calibration model, where healthy and tumor tissue samples are coded numerically (0 and 1, respectively). A new sample is assigned according to the predicted value with regard to the boundary of 0.5. The obtained values of sensitivity, selectivity, and accuracy for the most conservative SCV method indicate the general suitability of LED NIR-OMS for tumor recognition (Table 6) and the feasibility of further development of the system. The main problem remains the lack of sensitivity caused by large sample variability for the tumor tissue, which can be overcome by using more representative clinical data in the future. In the course of development, the optical channel configuration (their number and working wavelengths), as well as the measurement geometry, must be carefully optimized. The measurement information content can be increased by expanding the spectral region or by adding other spectroscopic techniques.

**Figure 17:**
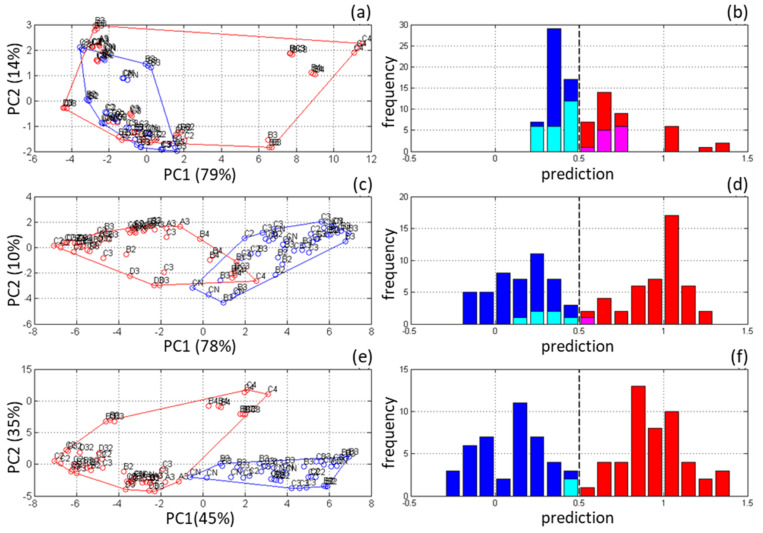
(**a**,**c**,**e**) PCA score plots and (**b**,**d**,**f**) prediction histograms for PLS models (SCV for measurement positions). Data used were: (**a**,**b**) fluorescence spectra, (**c**,**d**) IR-spectra, and (**e**,**f**) combined data. In (**a**,**c**,**e**) the red and blue colors designate the tumor and healthy tissue, respectively, and the labels indicate the measurement positions. In (**b**,**d**,**f**) the blue, cyan, red, and magenta designate *TN*, *FN*, *TP,* and *FP* results, respectively (see notes to Table 6).

The diagnostic capabilities of fluorescence and IR spectroscopy have been investigated in the second example, and their combination efficiency within one device has been proven [53]. To ensure the data compatibility, IR spectra (recorded on a FTIR-spectrometer with a PIR-fiber ATR probe) and fluorescence spectra (excited by a 473 nm laser and recorded through a diffuse reflectance probe) were recorded in the same marked positions (31 points in all 8 available samples). The resulting dataset included 92 pairs of IR and fluorescence spectra. The pairs were then analyzed both separately and jointly by concatenating them into one vector along the spectral axis. For the analysis, the IR spectra were limited to the most informative (from the point of view of the known biochemistry of the disease) region of 1220–1010 cm^−1^, and the fluorescence spectra, to the observed signal region at 490–680 nm. The optimal solution was sought using a combinatorial approach, which tested all the best preprocessing methods for each of the individual spectral blocks, as well as their various combinations in joint data analysis. The best preprocessing results selected on the basis of multilevel validation (Section 3) were chosen for the comparison of all three methods. Exploratory analysis showed a complete separation of the “cancer” and “normal” classes for the combined data only (Figure 17a,c,e). Notably, the models for both single and combined data required only two LVs. Maintaining the simplicity of the model while adding data is an indication of the complementarity of the combined blocks.

In the PLS-DA analysis (Figure 17b,d,f), the fluorimetry exhibits a lower discriminating ability than IR spectroscopy: their best accuracies *%Ac* according to the SCV results were 61 and 92, respectively. In the latter case, the incorrect classification is presented mainly by undesirable false negative results, i.e., by the unrecognized cancer (Figure 17d). Combining the fluorescence spectra with the second-derivative IR spectra followed by their standard normal variate (SNV) preprocessing leads to a decrease in false classifications to two (*%Ac* = 98, see Figure 17f). Therefore, the combination shows a synergistic effect. This fact makes it possible to suggest that the considered methods are responsible for various biomarkers. Moreover, due to the small penetration depth of IR spectroscopy, it “works” predominantly at the cellular level, while the fluorescent signal can come from a depth of several mm, carrying additional chemical and morphological information. This study confirms the feasibility of combining ATR-IR spectroscopy and fluorimetry in one analytical device [25]. When developing a multispectral analyzer, an IR spectrometer can be replaced by an OMS. Acquisition of fluorescence spectra can be significantly simplified, as well.

### 6.5. Environmental Monitoring

The final practical example is devoted to the development of an express method and OMS for the environmental monitoring of soils in order to determine the total petroleum hydrocarbons (TPH) using IR measurements through an ATR probe [100,101]. A preliminary analysis of the IR spectra was performed with a designed series of 57 artificial soil samples prepared by mixing a soil substrate, clay, sand, and dolomite flour. This study made it possible to investigate spectral properties of soils having various compositions and compare them with the spectra of crude oil. Based on this data, the spectral region of 4000–1700 cm^−1^ and the chalcogenide IR fiber ATR probe with a working crystal element of ZrO_2_ were found to be optimal for the quantitative analysis of TPH.

A calibration series of contaminated samples was prepared by adding oil obtained from the Mayorskoye field (Orenburg region, Russia) and water, as the main natural factors affecting the measurement, to 100 g of an artificial soil sample compositionally similar to the soil found in the Samara region (Russia). For the measurements, the probe was brought into close contact with the tableted sample, so that the crystal was completely immersed in it. The training set of 25 samples followed the diagonal scheme $d2_{0v1}^{25}$ (Figure 3b), in which both components varied in the range of 1–13%. This set was used to build calibration models for TPH and %H_2_O (g/100 g). The most accurate models were achieved using the method of interval optimization (Section 3). The following intervals were found to be optimal: 5 three-point intervals with averaging and without preprocessing for TPH and 4 individual variables after applying preprocessing methods for water. The *RMSE* values for TSV were, respectively: 1.1% for TPH and 0.6% for water at three LVs.

The main experimental problem of the method is the low overall spectral intensity of dry samples and poor measurement precision, which is generally typical for ATR analysis of solid materials. The proposed data preprocessing and analysis techniques largely overcome this negative influence, and the achieved determination errors are acceptable for a number of practical analytical tasks. The results obtained make it possible to recommend further development of OMS for the field determination of TPH based on IR spectroscopy in the range 4000–1700 cm^−1^ through a fiber-optic ATR probe. Further improvement of the method accuracy would require an improved measurement interface.

Therefore, a large experimental material presented in Section 6 has illustrated the principal feasibility of transfer from the full-spectrum measurement to OMS. The analysis can be carried out with minimum accuracy loss or even without it. The OMS development principles described in previous sections play an important role in achieving the best results. These are: DoE, the correct choice of the spectral method and measurement geometry; exploratory data analysis for a deeper understanding of objects and methods of analysis; mathematical optimization of optical channels of the future sensor; as well as the possible full usage of data-contained information at the modeling stage.

## 7. Conclusions

This work creates the scientific foundations for the development of specialized spectroscopic analyzers of low selectivity—optical multisensor systems, which represent a new trend in modern analytical spectroscopy. OMS has several unique utilitarian properties that significantly expand the analytical capabilities of optical spectroscopy compared to the traditional laboratory analysis. Some of the most useful features include: diminutiveness, portability, autonomous operation, in-line usability, and wide availability. Their anticipated widespread use for solving various problems of qualitative and quantitative analysis in industry, medicine, and other practical fields enhances the level of analytical control of many important aspects of human activity.

Many scientific and technical problems have been solved in this work: optimization of spectral analyzer for an application, training and validation of mathematical models, analytical process control, and others. A general OMS methodology has been proposed, including many practical recommendations for its development stages supported by real application examples.

The work provisions that can be applied in other branches of chemistry are of particular value from a scientific point of view. These include: the proposed principles of designing a multicomponent calibration experiment, the concept of a trajectory in the analytical process technology, and new approaches to the multivariate analysis of low-selectivity spectral data.

The presented results are of high practical importance in solving various problems of OMS development, improvement, and their practical application for field measurements, express analysis, and in-line monitoring of various objects and environments. With the increasing modern demand for chemical analysis, the role of the scientific approach to the development of multisensor systems will permanently increase.

## Figures and Tables

**Table 1:** Typical characteristics of OMS compared to traditional optical spectroscopy.

#	Parameter of Analyzer	Spectrometer	OMS
Usage			
1	Application area	universal	specialized
2	Autonomy	no	can be independent
3	Dimensions	desktop	miniature
4	Portability	stationary	portable
5	Place of analysis	installation site	arbitrary
6	User qualification	specialist	not required
7	Price	higher	lower
8	Technical maintenance	regularly required	minimal/not required
Technical features			
9	Selectivity	high	low
10	Number of channels	hundreds	a few
11	Spectral scale	uniform	individual
12	Spectral range	maximum wide	individual
13	Optical channels	resolved	can overlay
14	Resolution	maximal (excessive)	can be inapplicable
15	Measurement speed	up to milliseconds	up to microseconds
16	Device standardization	hardware	software
Math and software			
17	Application of chemometrics	recommended	required
18	Hardware and software	external	built-in
19	Prediction model	local	global

**Table 2:** The main stages and tasks of OMS development.

#	Stage	Tasks
1	Design	analysis of requirements, choice of spectroscopy method, full-spectrum measurements, channel optimization
2	Creation of prototype	loop: prototype creation/improvement—testing
3	Production	loop: production model improvement—testing
4	Commissioning	installation (if required), loop: building a preliminary prediction model—testing, building a working prediction model
5	Technical support	device diagnostics and support, model diagnostics, model improvement (local or global)

Note: Underlined steps are related to the application of chemometrics.

**Table 3:** Goals and tasks solved in PAT.

Goal	Task	Solution
Monitor	alarm row/end product quality	trajectory start/end point goes out of acceptability region
	aware normal process course	keep trajectory cursor within acceptability region
	determine end-point	trajectory cursor reaches predefined end criteria
	real time release	analytical space includes all critical quality attributes, the analytical cursor lag is minimized
Control	feed-back control	return deviated trajectory into the acceptability region
	feed-forward control	keep predicted trajectory within the product acceptability region
Understand	avoid technical risks	investigate trajectory features, study process effects
	avoid process analytical risks	use well-designed analytical space and high-quality trajectory
Optimize	reduce costs	design process with a shorter trajectory
	improve product quality	design analytical space for precise trajectory, restrict acceptability area

**Table 4:** Nested validation statistics of calibration PLS-models for different OMS prototypes in the analysis of milk for fat and total protein content.

	Method	OC ^a^	LV ^b^	*RMSE*			
				Cal.	LOOCV	SCV	TSV
Fat	Full-spectrum ^c^	401	5	0.089	0.098	0.102	0.103
	LED-OMS transmittance ^d^	7	5	0.088	0.095	0.099	0.090
	LED-OMS diffuse reflectance (ref.-free) ^e^	9	5	0.094	0.102	0.104	0.091
Protein	Full-spectrum	401	4	0.040	0.042	0.043	0.040
	LED-OMS transmittance	7	4	0.051	0.054	0.059	0.054
	LED-OMS diffuse reflectance (ref.-free)	7	5	0.065	0.071	0.073	0.072

Notes: ^a^ The number of optical channels; ^b^ the number of LVs in PLS-model; ^c^ diode-array spectrometer measurement in transmittance mode through a cuvette with optical path of 4 mm; ^d^ calculated LED-OMS in transmittance; optimal LEDs were chosen from a data base (emission maximum, nm): 400, 430, 450, 507, 831, 850, and 905; ^e^ calculated OMS for space-resolved measurement in diffuse reflectance mode; optimal parameters of calculated LEDs (maximum/half-height width, nm) at different channels were: 704/32, 973/54 (C_2_); 597/24, 616/26, 941/50 (C_3_); 516/20, 736/34, 852/42, 899/46 (C_5_) for fat and 489/18, 876/44 (C_1_); 419/14, 697/30 (C_4_); 490/18, 588/24 (C_5_); 439/16 (C_6_) for total protein analyzer.

**Table 5:** Nested validation statistics for the PLS calibration models for the determination of ethanol, glucose, and fructose built on a designed sample set using the data by different IR-OMS prototypes.

Set/Analyte		Spectral Method	Probe	LV ^a^	*RMSE*		
					Cal.	LOOCV	TSV ^b^
EG-set	ethanol	FTIR ^c^	DATR ^d^	2	4.55	5.79	5.10
		GrS ^e^	DATR	2	5.18	6.67	5.63
		GrS	LATR ^f^	2	7.76	10.58	8.34
		FPI ^g^	LATR	2	7.48	9.74	5.75
	glucose	FTIR	DATR	2	5.47	6.66	5.53
		GrS	DATR	2	9.77	11.99	10.87
		GrS	LATR	2	9.12	11.95	11.62
		FPI	LATR	2	8.30	9.87	5.96
FG-set	ethanol	FTIR	DATR	3	0.96	1.14	1.11
		GrS	DATR	3	2.21	3.82	3.71
		GrS	LATR	3	11.07	17.10	16.45
		FPI	LATR	3	2.92	6.64	1.58
	glucose	FTIR	DATR	3	2.35	2.92	1.82
		GrS	DATR	3	3.76	6.76	4.03
		GrS	LATR	3	7.78	14.11	9.30
		FPI	LATR	2	8.03	9.65	10.45

Notes: ^a^ The number of latent variables in PLS model; ^b^ test set according to DD (Figure 3b); ^c^ FTIR-spectrometer Matrix MF (full-spectrum method); ^d^ ATR probe with diamond element; ^e^ spectrometer prototype based on diffraction grid with pyroelectric detector; ^f^ ATR probe with the replaceable element formed by a PIR fiber loop; ^g^ tunable spectrometer-interferometer Fabry–Pérot.

**Table 6:** PLS-DA statistics for the diagnosis of kidney cancer by an LED-based OMS.

Dataset	Calibration ^a^							Segmented Cross-Validation ^b^						
	*TP* ^c^	*FP* ^d^	*TN* ^e^	*FN* ^f^	*Sn%* ^g^	*%Sp* ^h^	*%Ac* ^i^	*TP*	*FP*	*TN*	*FN*	*%Sn*	*%Sp*	*%Ac*
A33 ^j^	19	1	11	2	91	92	91	18	1	11	3	86	92	88
B170 ^k^	62	5	70	33	65	93	78	59	7	68	36	64	92	75
B140 ^l^	64	3	67	6	91	96	94	63	3	67	7	90	96	93

Notes: ^a^ All models are built with two LVs; ^b^ cross-validation by segments formed by individual measurement positions; ^c^ true positive (results) ^d^ false positive; ^e^ true negative; ^f^ false negative; ^g^ sensitivity %*Sn* = *100∙TP*/(*TP* + *FN*); ^h^ specificity %*Sp* = *100∙TN*/(*FP* + *TN*); ^i^ accuracy %*Ac* = 100∙ (*TP* + *TN*)/(*TP* + *FP* + *TN* + *FN*); ^j^ full data for the prototype A; ^k^ full data for the prototype B; ^l^ data of prototype B after outlier elimination: one sample and two measurement positions.

## Data Availability

Experimental data can be provided by the author on request.

## Abbreviations

2D	two-dimensional
API	active pharmaceutical ingredient
ATR	attenuated total reflection
CV	cross-validation
DAD	diode-array detector
DD	diagonal design
DoE	design of experiment
EEM	excitation-emission matrix
FN	false negative
FP	false positive
FPI	Fabry–Pérot interferometer
FTIR	Fourier-transform infrared
GA	genetic algorithm
iPLS	interval partial least squares
IR	infrared
LED	light-emitting diode
LOOCV	leave-one-out cross-validation
LV	latent variable
MCR	multivariate curve resolution
MLR	multiple linear regression
NIR	near infrared
OMS	optical multisensor system
PAT	process analytical technology
PHD	photo diode
PIR	polycrystalline infrared
PLS	projection on latent structures or partial least-squares
SNV	standard normal variate
TN	true negative
TP	true positive
TPH	total petroleum hydrocarbons
DA	discriminant analysis
PC	principal component
PCA	principal component analysis
RTR	real-time release
RMSE	root mean-square error
RMSEC	root mean-square error of calibration
RMSECV	root mean-square error of cross-validation
RMSEP	root mean-square error of prediction
SCV	segmented cross-validation
SPbSU	St. Petersburg State University
TSV	test-set validation
UV	ultraviolet
Vis	visible
